# Quality Characteristics of Raspberry Fruits from Dormancy Plants and Their Feasibility as Food Ingredients

**DOI:** 10.3390/foods12244443

**Published:** 2023-12-11

**Authors:** Sílvia Petronilho, Manuel A. Coimbra, Cláudia P. Passos

**Affiliations:** 1 Chemistry Research Centre-Vila Real, Department of Chemistry, University of Trás-os-Montes and Alto Douro, Quinta de Prados, 5001-801 Vila Real, Portugal; 2 Associated Laboratory for Green Chemistry (LAQV-REQUIMTE), Department of Chemistry, Campus Universitário de Santiago, University of Aveiro, 3810-193 Aveiro, Portugal; mac@ua.pt

**Keywords:** *Rubus idaeus*, drying, phenolic compounds, antioxidant activity, functional ingredients, bakery products, sensory properties

## Abstract

The raspberry (*Rubus idaeus* L.) is a soft red fruit consumed worldwide due to its bitter-sweet taste and phenolics-associated health benefits. During plant dormancy, raspberry fruits are discarded. However, this work hypothesised that these fruits have the chemical quality to be valorised, which would mitigate their waste if adequately stabilised. This can be achieved by drying. The Pacific Deluxe and Versailles varieties were dried by freeze- and convective-drying (30 °C and 40 °C). The freeze-dried fruits preserved their colour, drupelets structure, and phenolic content. Convective-drying promoted a significant fruit darkening, which was more evident at 30 °C due to the longer drying process, and a loss of drupelets structure. Both temperatures promoted a similar decrease in phenolic content, as determined by HPLC, although the ABTS^●+^ antioxidant activity at 40 °C was lower (IC_50_ = 9 compared to 13 μg AAE/mg dry weight). To incorporate dried raspberries into muffin formulations, while keeping their red colour, it was necessary to change the raising agent from sodium bicarbonate to baker’s yeast. Sensory analysis by a non-trained panel revealed good acceptance, showing that fresh or dried raspberry fruits from dormancy had suitable characteristics for use as food ingredients.

## 1. Introduction

The raspberry (*Rubus idaeus* L.) is a soft red fruit, consisting of a cluster of drupelets, consumed worldwide due to its characteristic bittersweet taste and beneficial effects, which are greatly linked to its phenolic-rich profile [1]. This fruit is produced by raspberry plants, which after fruiting undergo dormancy, a state of reduced metabolic activity occurring when the days shorten and the temperature cools down [2]. However, in contrast to wild populations of *R. idaeus* [3], raspberry plants in greenhouses still produce canes with lateral branches able to give flowers and fruits, although in lower yields (ca. 3 times lower) when compared to the harvest season [4]. These fruits are normally not harvested for commercial usage and are wasted during raspberry plant pruning. As a result of the great growth in raspberry fruit production in the last decade (ca. 75% since 2010, with a global production of ca. 896 thousand tonnes of fruits in 2020 [5]), several tonnes of fruits have been neglected from dormancy. Following the circular economy concept, these fruits can be used to obtain added-value products while allowing the minimisation of the output of this waste.

Raspberry fruits are considered to be a source of vitamin C [6], minerals, like iron and potassium [7], phenolic compounds, which are mainly anthocyanins [8], and dietary fibre [9]. As a result of their richness in these biomolecules, raspberry fruits have been related to different bioactive properties, including antioxidant, anti-inflammatory, and anti-cancer activities, among others [10].

Despite their popularity, fresh raspberry fruits are highly perishable due to their high moisture content, which limits the fruits’ shelf life to just a few days after harvest. To minimise fruit loss and to ensure its availability all year, raspberry fruits have been channelled to frozen storage or more shelf-stable processed products, such as juice, jam, or puree [11]. Nevertheless, dried fruit consumption is now highly valorised, and dried raspberry fruits are not an exception, being consumed as ready-to-eat snacks or incorporated into formulations of other foodstuffs, including cereal mixtures, and dairy and bakery products [12]. The impacts of different drying technologies on the colour, composition, and antioxidant properties of dried raspberry fruits have been explored, including solar and microwave drying [13], as well as combined drying methods such as osmotic dehydration with vacuum [14] and convective hot air drying [15], both of which are combined with the microwave drying. However, at the industrial level, the most popular procedures are still convective- and freeze-drying [16]. Previous studies have demonstrated that, when compared to convective-dried raspberry fruits, the freeze-dried ones better preserve the colour, nutritive value, and physicochemical composition of the fresh fruits [16,17]. Nevertheless, the influence of these drying technologies on the properties of raspberry fruits from a dormancy state has not yet been elucidated.

In this study, it was hypothesised that dried raspberry fruits harvested in their dormancy state have the same quality attributes as the commercially available fruits. Their drying allows their incorporation into bakery products as functional ingredients rich in phenolic compounds. To prove this hypothesis, two red raspberry varieties (Pacific Deluxe and Versailles) from the dormancy state were dried by freeze- and convective-drying. The influence of the drying techniques on the fruits’ characteristics in terms of phenolic composition, colour, and antioxidant activity was assessed. Then, the impact of the incorporation of these fruits in fresh and dried forms into muffin formulations was evaluated according to the chromatic, physical, and sensory properties.

## 2. Materials and Methods

### 2.1. Materials

Raspberry fruits from Pacific Deluxe and Versailles red primocane-fruiting varieties, both belonging to *Rubus idaeus* L., were provided by the Portuguese company *Framboesas da Graça* (Ponte de Vagos, Portugal). The red fruits were collected at the company greenhouses in December 2020 (winter season), during the plants’ dormancy state. The reagents and standards used were: NaOH (98.6%, Panreac, Barcelona, Spain), formic acid (99%, Chem-Lab, Zedelgem, Belgium), and acetonitrile (99.9%, Carlo Erba Reagents, Cornaredo, MI, Italy). Malvidin-3-glucoside (≥90%, HPLC), phenolphthalein, Folin–Ciocalteu reagent, sodium carbonate (≥99.5%), gallic acid (≥99%, HPLC), and ABTS (>98%) were from Sigma-Aldrich, Madrid, Spain. All the reagents used were of analytical grade.

### 2.2. Sampling

For each raspberry variety under study, ca. 1000 g of raspberry fruits was picked randomly from the company’s greenhouses, following a z-shaped pattern to avoid edge and centre effects. The samples were transported immediately under refrigeration (ca. 4 °C) to the laboratory where the raspberry fruits’ physicochemical parameters (fruit weight, pH, total soluble solids (°Brix), titratable acidity, and moisture content) were promptly determined [18]. The remaining fruits were stored at −20 °C for a maximum period of 1 week and then used for the drying processes and incorporation into muffins.

### 2.3. Raspberry Fruits Drying

For each variety, two drying processes were used, freeze-drying and convective-drying, and five independent analyses were carried out, in which the weight of each sample was recorded before and after drying. The frozen raspberry fruits were placed in a freeze-dryer (Labogene, Scanvac CoolSafe, Allerød, Denmark) for 48 h (P = 0.094 mbar; T = −49 °C). For the convective-drying process, the raspberry fruits were dried in a lab-scale convective oven (BINDER GmbH, Tuttlingen, Germany) at 30 °C and 40 °C to avoid the thermal degradation of the phenolic compounds [19]. For this, the frozen samples were placed in the oven with a constant airflow (speed at 100% for desired temperature accuracy maintenance), and the weight loss was monitored periodically in an analytical balance with 0.01 g to 0.001 g graduation (Marsden GF24 Balance Weighing Scale, Rotherham, UK) throughout the drying process: every 15 min for the 1st h; every 30 min in the next 2 h; and every 2 h and 4 h until the fruits reached ≤15% humidity, which is the benchmark to avoid microbiological contamination in dried fruits [20].

### 2.4. Raspberry Fruits Characterisation

#### 2.4.1. Physicochemical Parameters Determination

For each variety, ca. 20 g of fresh fruits was crushed, and the juice was vacuum filtered with 1.2 µm glass microfiber filters to remove any solid materials. Then, its pH, total soluble solids (°Brix), and titratable acidity were measured [18]. The pH of the juice was determined using a digital pH meter (micropH 2002, Crison, Barcelona, Spain). The total soluble solids were quantified using a portable refractometer (FG103/113, Zuzi, Auxilab S.L, Spain) with a reading scale of 0–40 ± 0.1 °Brix. The titratable acidity was performed according to the official method AOAC 942.15 [21] by titrimetry, using NaOH 0.1 M and phenolphthalein as the indicator. The moisture content of the fruits was determined by freeze-drying (Labogene, Scanvac CoolSafe, Allerød, Denmark) for 48 h (P = 0.094 mbar; T = −49 °C). Three independent replicates were used for each assay.

#### 2.4.2. Chromatic Properties

For both varieties, the chromatic properties of the fresh and dried fruits were assessed by tristimulus colorimetry (CIELab), using a portable colorimeter (Konica Minolta, CM-2600d/2500d) with a standard D8 light source and a visual angle of 8, calibrated using a white standard (L* = 94.61; a* = −0.53; b* = 3.62), following the supplier’s information. Herein, the CIELab coordinates L* (luminosity), b* (yellow/blue), and a* (red/green) were determined, as well as the total colour difference (ΔE) [22,23,24]. The assay was performed in triplicate, with 5 measurements taken per replicate.

#### 2.4.3. Total Phenolic Content

For each raspberry variety, ca. 20 g of fresh fruits was crushed in a mortar and the final volume (juice + pulp) was measured. For the dried samples, 50 mg was crushed and suspended in 1.5 mL of water to obtain the aqueous extract. Each suspension was centrifuged (5 min, 3000 rpm, room temperature) and filtered in a 0.45 µm nylon membrane to obtain clear juice/aqueous extracts. Then, the total phenolic content was determined, in triplicate, by the Folin–Ciocalteu method [25]. A calibration curve of gallic acid was built (20–250 μg/mL), and the absorbance was measured at 750 nm. The total phenolic content was expressed as μg of gallic acid equivalents (GAE)/mg of sample (dry weight, dw).

#### 2.4.4. Phenolic Profile Analysis

For the phenolic profile analysis, 1 mL of aqueous extracts from the fresh fruits/dried samples, obtained as described in Section 2.4.3, was used. Then, the recovered insoluble pellets obtained after centrifugation were submitted to a hydroalcoholic extraction with the addition of 1 mL of methanol. Both the aqueous and the methanolic extracts were filtered in a C_18_ column (6 mL, Discovery, Supelco, Algés, Portugal) [26]. After evaporation, both extracts were dissolved in distilled water, filtered (0.22 µm Nylon membrane), and analysed by ultra-high-performance liquid chromatography with a diode detector (UHPLC-DAD-ESI/MS^n^) (Ultimate 3000, Dionex Co./Thermo Scientific, Waltham, MA, USA). Analysis was conducted using a Hypersil Gold (Thermo Scientific, Waltham, MA, USA) C_18_ column (100 mm length; 2.1 mm i. d.; 1.9 μm particle diameter, end-capped) and an adaptation of the reported chromatographic separation conditions [27]. Briefly, eluent A—0.1% (*v*/*v*) formic acid and eluent (B) acetonitrile were used at an elution gradient of 5% to 40% of (B), at 14.7 min, and 100% (B) at 16.6 min, followed by the return to the initial conditions at 24 min, for a total run time of 34 min. The flow rate was 0.2 mL/min. The phenolic compounds were determined at 520 nm using their retention time and mass spectrum fragments (Table S1). A calibration curve using the anthocyanin malvidin-3-glucoside (0.06–6 mg/mL) was built [26], and the results from three independent aliquots were expressed as μg of malvidin-3-glucose equivalents (Mv 3-gE)/mg of sample (dw).

#### 2.4.5. Antioxidant Activity

The determination of the antioxidant activity, in the fresh and dried raspberry samples, was carried out using the ABTS [2,2′-azino-bis(3-ethylbenzothiazoline-6-sulphonic acid)] method [25]. A calibration curve for ascorbic acid (2–20 μg/mL) was built. The absorbance was read at 734 nm, and the results were expressed as IC_50_ (minimal sample concentration required to inhibit 50% of ABTS^●+^).

### 2.5. Muffin Preparation and Characterisation

#### 2.5.1. Preparation of the Muffin Formulations

The muffins were prepared according to a homemade recipe [28] (the ingredients can be seen in Table S2). To determine the content of raspberries to be used in the muffin dough, 60, 90, and 120 drupelets of fresh, convective-dried, and freeze-dried raspberries were incorporated into the final formulation. To evaluate the effect of the raising agent on the chromatic and sensory properties of the muffin formulations, sodium bicarbonate (chemical raising agent used in the original recipe) and yeast (natural raising agent) were used.

To prepare the muffins (Figure S1), unsalted butter was mixed with sugar in a mixer (Hoffen Food Expert LH6802) for 1 min at speed 2 using the hard dough tool. Then, the eggs were added, and the mixer speed was gradually increased to the maximum speed of 6 for 5 min. Finally, the flour and sodium bicarbonate were added and mixed at speed 6 for 5 min. Using an ice cream scoop (5.8 cm × 4 cm × 2.5 cm), the dough was placed in a silicone mould (diameter 6.5 cm × height 1.8 cm). After placing the dough in the mould, the raspberries were added, and the dough was uniformised again. This recipe allowed to obtain a yield of 22 muffins per baking. The muffins were placed in a previously heated (180 °C, 5 min) domestic oven (Zanussi Built In, Aveiro, Portugal) for 15 min at 180 °C. Each formulation was carried out in triplicate, and a negative control (without raspberries) was made. After being removed from the oven, the muffins were cooled down to room temperature, coated with aluminium foil, and stored for further characterisation.

#### 2.5.2. Characterisation of the Muffins

**Figure 1:**
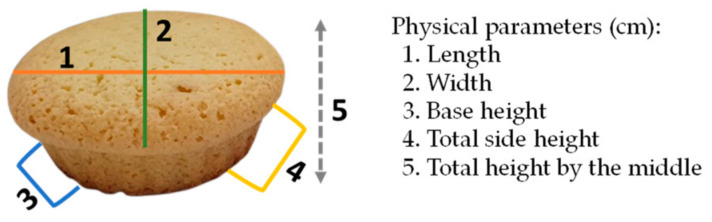
Scheme used to record the physical parameters of the muffins.

To understand the impact of the changes made to the original muffin formulation (incorporation of fresh, freeze-dried, and convective-dried raspberry fruits and raising agent), the dimension, weight, colour, and pH of the muffins were always evaluated 1 day after the muffins’ preparation. The physical parameters were recorded using a calliper, as shown in Figure 1. The weight was obtained using a kitchen scale (SilverCrest, Aveiro, Portugal) with a minimum capacity of 1 g and a maximum of 5 kg. Colour measurements were performed as described for the fresh and dried raspberries (Section 2.4.2), with 5 readings per replica (three independent replicates) on the upper and lower sides of the muffins.

The pH was determined based on an adaptation of a previous work [29]. Briefly, the samples removed from the middle of each cooked muffin (ca. 400 mg) were mixed with 20 mL of distilled water. The mixture was vortexed for 3 min and kept standing at room temperature for 1 h to separate the solid from the liquid phases. Then, the mixture was decanted and centrifuged (5 min, 4000 rpm). After centrifugation, the pH of the supernatants was measured using a digital pH meter (micropH 2002, Crison, Barcelona, Spain).

#### 2.5.3. Sensory Analysis of the Muffins

For sensory analysis, the muffin formulations containing yeast as the raising agent and a total of 120 drupelets of Versailles raspberries (fresh and freeze-dried) were used. The formulation without raspberries was used as the reference. The non-trained panel was composed of 5 males and 11 females, between the ages of 20 and 58, from the GlycoFoodChem research group at the University of Aveiro (Portugal). A hedonic scale test was used to measure the sensory quality of the muffins using 5 parameters: 1—dislike a lot, 2—dislike, 3—indifferent, 4—like, and 5—like a lot. The evaluation form given to the panellists listed different parameters (appearance, colour, aroma, sweetness, acidity, saltiness, fruitiness, global taste, softness, hardness, global texture, and global appreciation) and score options with number rankings from 1 to 5. The samples were presented in random order and coded: muffins without raspberries (CN), muffins with 120 fresh raspberry drupelets (MF), and muffins with 120 freeze-dried raspberry drupelets (ML).

### 2.6. Statistical Analysis

The data were statistically analysed by Student’s *t*-test with a level of significant difference of 95% and *p* < 0.05, using the “test *t*” tool of Excel 2016. For multiple comparison analysis, the significance of the difference was evaluated with one-way ANOVA at the significance level of *p* < 0.05, followed by Tukey’s multiple comparison test using GraphPad Prism 5.01 software (OriginLab Corporation, Northampton, MA, USA, trial version).

## 3. Results and Discussion

### 3.1. Fresh Raspberry Fruits Physicochemical Characterisation

The fresh fruits from the Pacific Deluxe dormancy had a weight of 5.73 g per fruit, and their juice had a pH of 3.25, a °Brix of 5.10, and a titratable acidity of 0.90% citric acid equivalents (CAE)/100 mL of juice (Table 1). When compared to the Pacific Deluxe fruits, the Versailles fruits had a higher weight (6.96 g per fruit), and the resulting juice had higher acidity (pH of 2.86 and titratable acidity of 1.25% CAE/100 mL) and sweetness (°Brix of 9.07). The moisture content of the Pacific Deluxe (90.21%) and Versailles (86.98%) fruits was also significantly different. The Versailles fruits seem to be appropriate for conferring acidity and sweetness if used in juices, when compared to the Pacific Deluxe fruits. Despite this variability, the physicochemical parameter values, except for those of the total solids of Pacific Deluxe, were within the range reported for the other *R. idaeus* species collected at commercial maturity: pH of 2.9 to 4.2, total solids of 5.5 to 12.6 °Brix, titratable acidity of 0.8 to 1.9 of CAE/100 mL of juice, and moisture of 82.0 to 90.3% [12,13,17,30,31,32]. These results allowed the inference that fruits from the dormancy state have similar physicochemical attributes to those of the commercially available fruits.

### 3.2. Raspberry Fruit Drying

**Figure 2:**
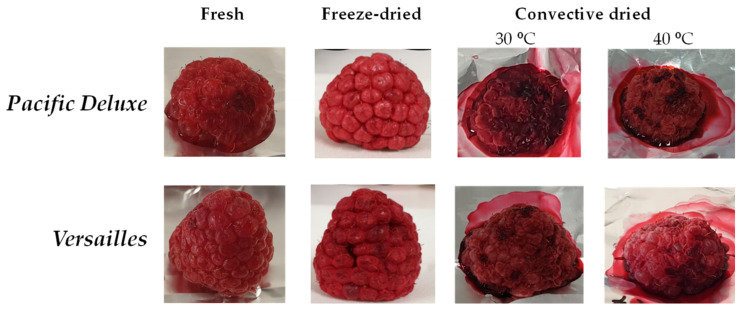
Images of fresh, freeze-dried, and convective-dried (30 °C and 40 °C) raspberry fruits from dormancy state of Pacific Deluxe and Versailles varieties.

The freeze-drying (−49 °C, 48 h) of the Pacific Deluxe and Versailles fruits resulted in 0.56 and 0.83 g per fruit, respectively, which is consistent with the larger fruit size and lower moisture of the Versailles variety (Table 1). The structured cluster of the drupelets of the fresh fruits was kept when dried, as was the ruby colour (Figure 2).

The convective-drying of raspberry fruits was performed at 30 °C and 40 °C (Figure 2) since these drying temperatures promote the thermal degradation of the phenolic compounds and the antioxidant activity of red fruits, like raspberries from the Autumn Bliss variety [26] and other non-specified *R. ideaus* varieties [17].

**Figure 3:**
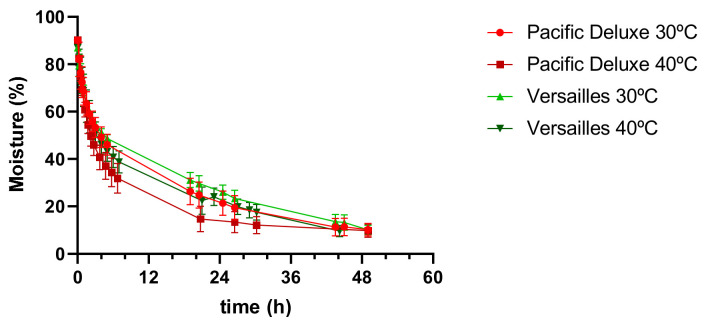
Drying curves of raspberry fruits from dormancy state of Pacific Deluxe and Versailles varieties, obtained at 30 °C and 40 °C.

The drying process was followed through the construction of the drying curves as a function of time until reaching a moisture content of ≤10% (Figure 3), which is below the 15% benchmark to avoid microbial contamination [20]. It took around 49 h at 30 °C for both varieties and ca. 10% less (44 h) at 40 °C, which is a decrease that shows the same trend as that of the commercially available raspberries dried at 50 °C, 65 °C, and 130 °C [16]. In contrast to the freeze-dried fruits, the convective-dried fruits acquired a non-uniform dark colour and had volumetric shrinkage and a change in shape, losing their drupelets cluster structure (Figure 2). This was not so evident for the Versailles variety, probably due to their higher weight and total solids content (Table 1).

### 3.3. Impact of the Drying Processes

#### 3.3.1. Raspberry Fruit Colour

The fresh raspberries from Pacific Deluxe had L*, a*, and b* values of 33.35, 29.16, and 12.78, respectively, reflecting a ruby colour, as represented in Table 2 [33]. When compared to the fresh fruits, the freeze-dried ones had significantly higher a* (35.74) and lower b* (10.85) values, which can be translated into a more intense red colour with less yellow. This was explained as being a consequence of the more efficient light diffusion through the raspberry fruit due to the free water replacement by air during freeze-drying [16].

The raspberries dried at 30 °C presented a significant decrease in all the chromatic parameters (L* = 20.17, a* = 22.02, b* = 7.57) when compared to the fresh fruits, reflecting the darkness and browning of the ruby colour. However, this darkness was less intense when the fruits were dried at 40 °C, with L* and b* values of 25.01 and 11.50, and no significant changes in the red–green (a*) coordinate. These results were consistent with the literature for the commercial raspberry fruits dried at 50 °C [26]. The colour darkening can be explained by a possible decomposition of the carotenoids and/or non-enzymatic browning related to Maillard reactions, which resulted in the formation of brown compounds [19]. The total colour difference (∆E) achieved for the freeze-dried (∆E = 6.89) and convective-dried fruits (30 °C—∆E = 15.9 and 40 °C—∆E = 8.44) corroborated the discussed chromatic changes. However, only the ∆E value for the Pacific Deluxe fruits dried at 30 °C was beyond 10, the benchmark used to consider a significant colour degradation in dried fruits [16]. The greater darkening of Pacific Deluxe dried at 30 °C (Figure 2, Table 2) suggested that when the drying time was higher (49 h) the extension of the browning reactions would be higher.

For the Versailles fresh fruits, the obtained L*, a*, and b* values were 32.86, 31.98, and 13.42, respectively. These values are not significantly different from those of Pacific Deluxe. The freeze-dried fruits exhibited a significant decrease in their luminosity (L* = 25.55), and no significant changes were observed for the a* and b* coordinates. A similar trend was observed for the fruits dried at 30 °C and 40 °C. The ∆E values varied from 5.16 to 8.28, which were values below 10, thus suggesting no significant degradation of the ruby colour of the dried Versailles fruits [16], as with the Pacific Deluxe samples freeze-dried and dried at 40 °C (Table 2).

#### 3.3.2. Raspberry Fruit Phenolics and Antioxidant Activity

**Figure 4:**
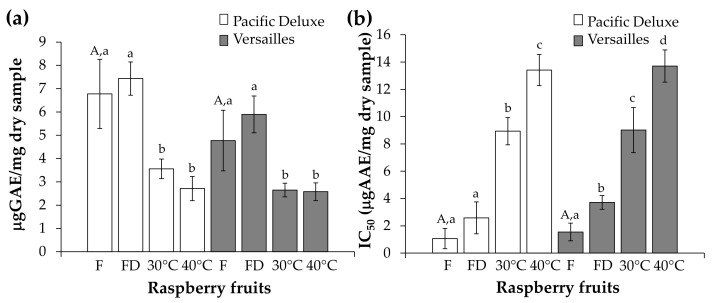
Total phenolic content (**a**) and antioxidant activity (**b**) of fresh (F), freeze-dried (FD), and convective-dried raspberry fruits from dormancy state at 30 °C and 40 °C. The same uppercase letter represents no significantly different values between fresh fruits (*p* > 0.05). For each variety, different lowercase letters represent significantly different values (*p* < 0.05). GAE—gallic acid equivalents and AAE—ascorbic acid equivalents.

The fresh Pacific Deluxe fruits presented 6.78 µg GAE/mg (dw) of phenolic compounds, as determined by the Folin–Ciocalteu method (Figure 4a), which was in line with the literature concerning other red raspberry varieties [34]. After freeze-drying, no significant changes in the total phenolics content of the raspberry fruits were observed. However, after convective-drying, a significant decrease was determined (3.56 and 2.71 µg GAE/mg (dw) at 30 °C and 40 °C, respectively), although it was not statistically different between the two drying temperatures used. These results revealed a decrease of ca. 48-60% of the phenolics amount when applying convective-drying to the raspberry fruits. A similar trend was observed for the Versailles fruits: no significant changes were determined for the fresh and freeze-dried fruits (4.77 and 5.89 µg GAE/mg (dw), respectively), while a decrease of ca. 45% was registered for the fruits dried at 30 °C and 40 °C (phenolic content of 2.65 and 2.58 µg GAE/mg (dw), respectively) (Figure 4a). These results were in line with literature reports on other *R. ideaus* varieties, where drying temperatures of 50 °C [16,17] and 60 °C [12] led to a decrease in the raspberry fruits’ phenolic content between ca. 30 and 63%, while no remarkable changes were observed in the freeze-dried fruits. Beyond temperature, the significant decrease in the total phenolic content during convective-drying can be related to the prolonged drying times required when temperatures are mild [16], which is probably due to the promotion of enzymatic activity able to release phenolics linked to carbohydrates [26], which are more prone to oxidisation [35].

The phenolic profile of the fresh, freeze-dried, and convective-dried raspberry fruits from the Pacific Deluxe and Versailles varieties is presented in Table 3 (and there is an example of a chromatogram in Figure S2). A total of four anthocyanins were determined in both varieties, with cyanidin-3-*O*-sophoroside being the most abundant one. This profile was in line with the literature [26] and reflects the anthocyanins’ compositional homogeneity among the red varieties studied.

The fresh fruits from Pacific Deluxe had a total anthocyanin content of 11.52 μg malvidin-3-glucose equivalents (Mv 3-gE)/mg (dw), where ca. 65% corresponded to cyanidin-3-*O*-sophoroside (Cy 3-s = 7.54 μg Mv 3-gE/mg, dw). When compared to the fresh fruits, a similar content and profile was obtained for the freeze-dried Pacific Deluxe samples, while a significant decrease in the total amount of anthocyanins was determined in the convective-dried fruits (a decrease of ca. 14% and 30% in the fruits dried at 30 °C and 40 °C, respectively). This was even more evident for Cy 3-s, where a decrease from 7.54 to 4.90 μg Mv 3-gE/mg (dw) was observed in the fruits dried at 40 °C (Table 3). This followed the trend observed in commercially available *Rubus idaeus* L. raspberry fruits when submitted to drying at 50 °C for 19 h, where Cy-3-s decreased by ca. 78% [17]. This trend was consistent with the results determined by the Folin–Ciocalteu method (Figure 4a), although the decrease percentages obtained by HPLC analysis were lower than those of the colorimetric method, suggesting that, beyond anthocyanins, other phenolics were degraded during the convective-drying.

The fresh Versailles raspberry fruits presented a total anthocyanin content of 10.63 μg Mv 3-gE/mg (dw), which was significantly lower than that of the Pacific Deluxe fresh fruits, except for cyanidin-3-(6’-succinyl-glucoside), which was ca. 2.7 times higher in the Versailles fruits. When compared to the Versailles fresh fruits, no significant changes were determined in the freeze-dried ones (11.51 μg Mv 3-gE/mg, dw), while a decrease of ca. 37% and 44% was reached for the fruits dried at 30 °C and 40 °C, respectively (Table 3), according to the trend observed with the Folin–Ciocalteu method (Figure 4a). These results suggested that for the Versailles fruits the ca. 45% reduction in the total amount of phenolics can be mainly related to the possible degradation of anthocyanins, which is in line with the literature [12,17].

The antioxidant activity of the raspberry fruits from Pacific Deluxe and Versailles was evaluated by determining the samples’ ability to scavenge the ABTS^●+^ radical (Figure 4b). Regarding the antiradical activity, the concentration of fresh Pacific Deluxe fruits able to inhibit 50% of ABTS (IC_50_) was 1.07 μg ascorbic acid equivalents (AAE)/mg (dw). The freeze-dried fruits had an IC_50_ of 2.58 μg AAE/mg (dw), which was not significantly different from that of the fresh fruits. Moreover, IC_50_ values of 8.94 and 13.41 μg AAE/mg (dw) were determined for the convective-dried fruits at 30 °C and 40 °C, respectively; these were significantly higher than those for the fresh fruits. The increase in these values can be translated into a significant decrease in the antiradical activity of the convective-dried fruits. This effect was more evident for the higher temperature tested, where the IC_50_ value was ca. 13 times higher than that of the fresh fruits and thus presented lower antioxidant activity (Figure 4b).

The fresh fruits of the Versailles variety presented an IC_50_ = 1.55 μg AAE/mg (dw), which was not significantly different from that of the Pacific Deluxe fresh fruits. When compared to the fresh fruits, all the Versailles dried fruits exhibited a significant decrease in their antiradical activity. The drying at 40 °C was the procedure that had the higher impact on the decrease in these fruits’ antioxidant activities (Figure 4b), which were similar to those of the Pacific Deluxe fruits. These results were consistent with those of the literature, where the antioxidant activity of commercial fresh red raspberry fruits (*R. idaeus* cv. Heritage) was significantly higher than that observed in fruits dried by microwave and solar drying processes [13].

As the antioxidant activity is largely related to the content of phenolic compounds, the higher IC_50_ values observed in the convective-dried samples can be explained by the lower phenolic content registered. As the content of total phenolic compounds determined for the dried raspberries at 30 °C and 40 °C was very similar (Figure 4a), the significant differences in the antioxidant activity suggested that there are other compounds with antioxidant potential that also decrease their activity during drying. This was more evident when the process was carried out at 40 °C (Figure 4b). This may be due to the high content of ascorbic acid in raspberry fruits, which is a compound sensitive to thermal treatments [16].

### 3.4. Muffin Development

Fresh, freeze-dried, and convective-dried (40 °C) Versailles fruits from dormancy were used as ingredients for the development of muffins. The Versailles variety was selected since lower chromatic changes and a similar phenolic content were observed during drying when compared to the Pacific Deluxe dried fruits (Figure 2, Table 2). Drying at 40 °C was selected instead of 30 °C because no significant changes were observed in the phenolic content of the fruits (Figure 4a), and a 10% reduction in the drying time was achieved (Figure 3); this is a more sustainable option that can allow a decreasing of the processing energy costs.

For muffin production, the content of raspberry drupelets (60, 90, and 120 drupelets) to be included in the muffin dough was optimised. The impact of the fruits on the physical properties, colour, and sensory attributes of the muffins was evaluated, and the selection of the raising agent (chemical—sodium bicarbonate or natural—yeast) was performed. A baking negative control was always carried out, and it corresponded to the formulation made without the incorporation of raspberry fruits.

#### 3.4.1. Influence of Drupelets Incorporation on Physical Properties and Colour of Muffins

Different quantities of fresh, freeze-dried, and convective-dried (40 °C) raspberry fruits (60, 90, and 120 drupelets) were incorporated in a muffin formulation made with sodium bicarbonate as the raising agent (original recipe). The physical properties of the muffins were evaluated to assess the influence of the number of drupelets on the muffins’ dimensions (Table 4). When looking at the weight of the muffins, with the incorporation of fresh fruit drupelets in the formulations, a significant increase in weight was observed, ranging from 38.3 g in the muffins without drupelets to a maximum of 51.7 g in the muffins containing 120 fresh drupelets. The muffins’ width (parameter 2) also increased, reaching its maximum at 9.1 cm for the muffins with 120 drupelets. According to these results, the muffins became heavier and larger with the increase in the number of fruit drupelets, probably due to the higher water content of the fresh fruits. This may be related to higher water activity and thus should be considered when looking at the microbiological stability of this baked product. No significant changes were observed in the remaining parameters (1—length, 3—base height, 4—total side height, and 5—total height in the middle).

With the incorporation of freeze-dried fruits, in contrast to the use of fresh fruits, a significant decrease in the weight of the muffins was observed, from 38.3 g in the muffins without drupelets, to 36.0 and 34.7 g in the muffins containing 90 and 120 drupelets, respectively. No significant differences were observed among the muffins with these two quantities. Also, the length (parameter 1) and total height in the middle (parameter 5) significantly decreased for all the tested drupelets contents, but no significant changes were observed among the different quantities. These results revealed that the muffins with the freeze-dried fruits became smaller, which was more relevant for the muffins with 90 and 120 drupelets. The lower moisture of these freeze-dried fruits (Table 1) may explain the smaller size of the resulting muffins (Table 4). The opposite was observed in the muffins with the convective-dried fruits, where only the muffins with 120 drupelets became heavier (46.0 g). No significant changes were observed for the other physical parameters (from 1 to 5). In this case, the reduced moisture content of the fruits dried at 40 °C (≤10%, Figure 3) may explain the muffins’ higher weight when using a higher number of drupelets, which is like the weight of the muffins containing 90 drupelets of fresh fruits and lower than the weight of the muffins with 120 fresh drupelets (Table 4).

The influence of the fruit incorporation on the colour properties of the muffins on the upper (Table 5) and lower sides (Table S3) showed that the upper side of the muffins, with the incorporation of fresh fruit drupelets in the formulations, significantly decreased in all chromatic values (L*, a*, and b*) when compared to the corresponding control (muffins without drupelets). These differences were not significant among the concentrations of drupelets used. This was manifested in the darkening of the muffins and in the appearance of a greenish colour. The ΔE > 10 for all the assays with the fresh fruits corroborated the significant chromatic changes. With the incorporation of the convective-dried samples, only a significant decrease in the a* value was observed, which reflected the appearance of a greenish colour in the muffins. Although this difference was not significant among the drupelets amounts used, all the ΔE > 10 revealed a significant colour change. This effect was not observed in the muffins with the freeze-dried samples. In this case, for all the tested drupelets amounts, the L*, a*, and b* values were similar to those of the control (L* = 66.06, a* = 6.07, b* = 38.34), and the ΔE was below 5, which was ca. 2 times lower than the ΔE benchmark for a colour change to be considered significant [16]. When analysing the lower side of the muffins, no significant changes were determined for all the tested conditions (Table S3). The visual aspect of the muffins corroborated these results (Figure S3).

The major colour changes observed in the muffins made with fresh raspberry drupelets, followed by the muffins containing convective-dried fruits, can be related to the release of moisture contained in the drupelets during cooking, a phenomenon known as syneresis [36,37]. The release of water from the drupelets also led to the release of soluble phenolic compounds, namely anthocyanins, which at the pH of the dough (ca. 10 for all tested formulations, independently of the drupelets amount) gave a greenish colour to the muffins [38]. The syneresis effect was greater as the moisture content of the sample becomes greater, which is the reason why fresh drupelets provoked higher colour changes in the muffins than the ones dried at 40 °C (moisture content ca. 10%), and no changes were observed in the muffins with freeze-dried fruits.

#### 3.4.2. Influence of the Raising Agent on the Colour and Physical Properties of the Muffins

The green colour of the muffins made with fresh and dried raspberry fruits at 40 °C did not make the final greenish colour of the product attractive. For that reason, it was decided to replace the sodium bicarbonate with baker’s yeast, a natural raising agent. The impact of this replacement on the pH of the dough and the resulting muffins’ colour and physical properties was evaluated. This assay was made only in muffins containing 120 fresh drupelets, where the green colour was strongly evidenced (ΔE = 18.06, Table 5).

When replacing the sodium bicarbonate with yeast, the muffin dough containing fresh raspberry drupelets lost its green colour and exhibited a light-yellow colour (Figure S4a). The ΔE of 4.91 and 2.61 determined on the upper and lower sides of these muffins, respectively, corroborated their colour similarity with the muffins with zero drupelets (Table 6). Due to the raising agent replacement, the pH of the muffin dough changed from ca. 10 (sodium bicarbonate raising agent) to ca. 7 in the muffins made with yeast (Figure S4b). This colour change is related to the reversible structural transformations that anthocyanins undergo when the pH values of the environment change [39,40]. At pH 7, the anthocyanin structure is protonated, presenting a purple quinoid anhydrous base [41,42]. Therefore, it is possible to conclude that the green colour of the muffins containing raspberry drupelets was avoided by replacing the sodium bicarbonate with yeast. Additionally, the substitution of the raising agent resulted in heavier muffins with no significant changes in the other dimensions (Table S4). Based on these results, yeast was used in the recipe for further sensory analysis of the muffins.

#### 3.4.3. Sensory Analysis of Muffins

**Figure 5:**
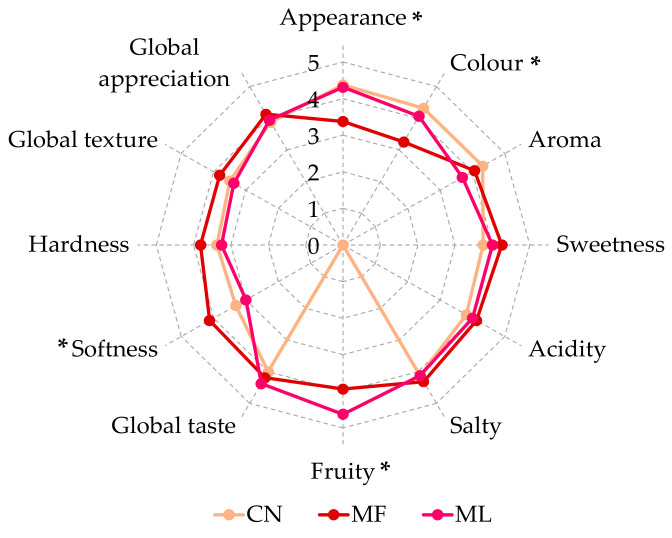
Sensory data, expressed as mean values, of muffins without raspberries (CN), with 120 fresh raspberry drupelets (MF), and with 120 freeze-dried raspberry drupelets (ML), based on the 12 sensory terms used by the non-trained panel (16 panellists). * *p* < 0.05.

To evaluate the differences that can be perceived by the incorporation of raspberries in a fresh or dried state, freeze-dried fruits were used to prepare the muffin recipes using the baker’s yeast as the raising agent and a total of 120 drupelets of Versailles raspberries (fresh—MF and freeze-dried—ML). The muffins without fruits were also considered (CN). A five-parameter hedonic scale was used by the 16 non-trained panellists. The global appreciation (ranging from 3.9 and 4.1), as well as the global texture (ranging from 3.4 and 3.8), showed that all the products were well appreciated by the panellists (Figure 5).

The muffins with freeze-dried fruits were significantly better appreciated than the muffins with fresh fruits with regard to their appearance and colour. However, no significant differences were observed for the control, allowing the inference that the incorporation of dried fruits in the formulation is an advantage when compared to the fresh ones. Nevertheless, the muffins prepared with fresh fruits presented significantly higher softness than those prepared with dried fruits and the control.

## 4. Conclusions

The raspberry fruits harvested during the dormancy state of the plant showed similar characteristics to those of the commercially available fruits, as referred to in the literature. In the studied case regarding the physicochemical differences between the Pacific Deluxe and Versailles fruits, the structure and composition of the fresh fruits were quite similar when the fruits were freeze-dried or when they were submitted to convective-drying at 30 °C or 40 °C. In both cases, the freeze-drying allowed better maintenance of the ruby colour and the cluster of drupelets of the fresh fruits, as well as their total phenolic content and antioxidant properties. Nevertheless, convective-drying at 40 °C, although presenting a darker colour and a lower antioxidant activity than the fresh and freeze-dried fruits, presented properties that allowed them to also be considered as food ingredients. These fruits can be used in muffin formulations. For that, the selection of the raising agent is of major relevance, as raspberry anthocyanins tend to be green due to the alkaline pH of the dough when using sodium bicarbonate. To overcome this drawback, baker’s yeast was shown to be an effective substitute. The sensory analysis performed by the non-trained panel attested to the global appreciation and acceptance of the muffins containing dried fruits, showing that raspberry fruits from dormant plants have quality characteristics and that it is feasible to use them as food ingredients.

## Figures and Tables

**Table 1:** Physicochemical characterisation of fresh raspberry fruits from dormancy state of Pacific Deluxe and Versailles red varieties.

Variety	Weight (g) ^1^	pH	°Brix	Titratable Acidity (% CAE/100mL) ^2^	Moisture (%) ^3^
Pacific Deluxe	5.73 ± 0.04 ^a^	3.25 ± 0.14 ^a^	5.10 ± 0.17 ^a^	0.90 ± 0.02 ^a^	90.21 ± 1.13 ^a^
Versailles	6.96 ± 0.26 ^b^	2.86 ± 0.06 ^b^	9.07 ± 0.12 ^b^	1.25 ± 0.03 ^b^	86.98 ± 1.58 ^b^

^1^ Values per berry. ^2^ CAE—citric acid equivalents. ^3^ Moisture content determined after freeze-drying. Data presented are expressed as the mean and standard deviation of three independent replicates. In each column, different lowercase letters represent significantly different values (*p* < 0.05).

**Table 2:** Mean values of lightness (L*), red–green (a*), yellow–blue (b*), and total colour difference (ΔE) of fresh, freeze-dried, and convective-dried (30 °C and 40 °C) raspberry fruits from dormancy state of Pacific Deluxe and Versailles varieties.

	L*	a*	b*	ΔE	Colour ^1^
**Pacific Deluxe**					
Fresh	33.35 ± 0.83 ^A,a^	29.16 ± 4.26 ^A,a^	12.78 ± 1.71 ^A,a^	-	
Freeze-dried	32.68 ± 2.53 ^a^	35.74 ± 2.64 ^b^	10.85 ± 0.94 ^b^	6.89	
Dried at 30 °C	20.17 ± 1.81 ^b^	22.02 ± 4.77 ^c^	7.57 ± 2.12 ^c^	15.87	
Dried at 40 °C	25.01 ± 3.38 ^c^	29.05 ± 1.66 ^a^	11.50 ± 0.97 ^b^	8.44	
**Versailles**					
Fresh	32.86 ± 1.39 ^A,a^	31.98 ± 2.32 ^A,a^	13.42 ± 1.93 ^A,a^	-	
Freeze-dried	25.55 ± 2.08 ^b^	34.68 ± 3.05 ^a^	11.73 ± 1.20 ^a^	7.98	
Dried at 30 °C	25.03 ± 4.45 ^b^	29.82 ± 6.33 ^a^	11.85 ± 3.39 ^a^	8.28	
Dried at 40 °C	28.15 ± 3.72 ^b^	30.52 ± 3.50 ^a^	11.90 ± 1.73 ^a^	5.16	

^1^ Fruits colour using an online correspondence CIELab parameters program using RGB (red, green, and blue) values [33]. The same uppercase letter represents no significantly different values between fresh fruits (*p* > 0.05). For each variety, in each column, different lowercase letters represent significantly different values (*p* < 0.05).

**Table 3:** Phenolic compounds (μg Mv 3-gE/mg sample, dw) determined in fresh, freeze-dried, and convective-dried (30 °C and 40 °C) raspberry fruits from dormancy state of Pacific Deluxe and Versailles varieties.

	Cy 3-s	Cy 3-gr	Cy 3-(6”-dg)	Cy 3-(6”-s-gl)	Total
**Pacific Deluxe**					
Fresh	7.54 ± 0.36 ^A,a^	2.52 ± 0.08 ^A,a^	1.00 ± 0.08 ^A,a^	0.46 ± 0.02 ^A,a^	11.52 ± 0.38 ^A,a^
Freeze-dried	8.01 ± 0.79 ^a^	2.87 ± 0.34 ^a^	1.10 ± 0.10 ^a^	0.53 ± 0.04 ^a^	12.52 ± 1.29 ^a^
Dried at 30 °C	6.29 ± 0.11 ^b^	2.18 ± 0.09 ^b^	0.92 ± 0.03 ^b^	0.47 ± 0.01 ^a^	9.86 ± 0.16 ^b^
Dried at 40 °C	4.90 ± 0.58 ^c^	1.99 ± 0.27 ^b^	0.76 ± 0.05 ^c^	0.41 ± 0.04 ^a^	8.06 ± 0.49 ^c^
**Versailles**					
Fresh	5.41 ± 0.10 ^B,a^	2.82 ± 0.12 ^B,a^	1.18 ± 0.05 ^B,a^	1.22 ± 0.07 ^B,a^	10.63 ± 0.05 ^B,a^
Freeze-dried	5.84 ± 0.24 ^a^	3.07 ± 0.11 ^a^	1.23 ± 0.03 ^a^	1.37 ± 0.09 ^a^	11.51 ± 0.57 ^a^
Dried at 30 °C	3.25 ± 0.11 ^b^	1.73 ± 0.24 ^b^	0.93 ± 0.21 ^b^	0.84 ± 0.11 ^b^	6.75 ± 0.23 ^b^
Dried at 40 °C	2.95 ± 0.07 ^c^	1.46 ± 0.18 ^b^	0.81 ± 0.18 ^b^	0.70 ± 0.09 ^b^	5.92 ± 0.13 ^c^

Cy-3-s: Cyanidin-3-O-sophoroside; Cy-3-gr: Cyanidin-3-O-glucosyl rutinoside; Cy 3-(6”-dg): Cyanidin 3 (6’-dioxalyl-glucoside); Cy 3-(6”-s-gl): Cyanidin-3-(6’-succinyl-glucoside); Mv 3-gE: Malvidin-3-glucose equivalents. Different uppercase letters represent significantly different values between fresh fruits (*p* > 0.05). For each variety, different lowercase letters represent significantly different values (*p* < 0.05).

**Table 4:** Physical parameters of muffins baked with different drupelets amounts (0, 60, 90, 120) of fresh, freeze-dried, and convective-dried (40 °C) Versailles raspberry fruits from dormancy state.

			Parameters (cm)				
	Drupelets	Weight (g)	1	2	3	4	5
Fresh	0	38.3 ± 2.0 ^a^	6.8 ± 0. 1^a^	8.3 ± 0.4 ^a^	1.5 ± 0.2 ^a^	2.6 ± 0.5 ^a^	2.7 ± 0.0 ^a^
	60	41.3 ± 2.1 ^a^	7.4 ± 0.5 ^a^	8.2 ± 0.2 ^a^	1.5 ± 0.2 ^a^	2.7 ± 0.3 ^a^	2.7 ± 0.0 ^a^
	90	46.3 ± 2.1 ^b^	7.4 ± 0.5 ^a^	8.8 ± 0.2 ^b^	1.5 ± 0.2 ^a^	2.6 ± 0.1 ^a^	2.7 ± 0.0 ^a^
	120	51.7 ± 1.5 ^c^	7.3 ± 0.3 ^a^	9.1 ± 0.2 ^b^	1.5 ± 0.1 ^a^	2.5 ± 0.2 ^a^	2.8 ± 0.1 ^a^
Freeze-dried	0	38.3 ± 2.1 ^a^	6.8 ± 0.1 ^a^	8.3 ± 0.4 ^a^	1.5 ± 0.2 ^a^	2.6 ± 0.5 ^a^	2.7 ± 0.0 ^a^
	60	38.0 ± 1.0 ^a^	7.7 ± 0.2 ^b^	7.8 ± 0.2 ^a^	1.5 ± 0.1 ^a^	2.4 ± 0.1 ^a^	2.2 ± 0.2 ^b^
	90	36.0 ± 1.5 ^b^	7.8 ± 0.5 ^b^	8.3 ± 0.3 ^a^	1.5 ± 0.2 ^a^	2.6 ± 0.3 ^a^	1.9 ± 0.2 ^b^
	120	34.7 ± 1.0 ^b^	7.3 ± 0.2 ^b^	7.9 ± 0.2 ^a^	1.5 ± 0.1 ^a^	2.6 ± 0.1 ^a^	1.9 ± 0.2 ^b^
Dried at 40 °C	0	38.3 ± 3.1 ^a^	7.7 ± 0.3 ^a^	8.6 ± 0.2 ^a^	1.5 ± 0.0 ^a^	2.8 ± 0.3 ^a^	2.0 ± 0.1 ^a^
	60	42.3 ± 2.1 ^a^	8.9 ± 1.0 ^a^	9.0 ± 0.4 ^a^	1.6 ± 0.2 ^a^	2.9 ± 0.2 ^a^	1.9 ± 0.0 ^a^
	90	41.7 ± 2.5 ^a^	7.8 ± 0.7 ^a^	9.1 ± 0.2 ^a^	1.4 ± 0.1 ^a^	2.7 ± 0.2 ^a^	1.8 ± 0.0 ^a^
	120	46.0 ± 1.0 ^b^	8.1 ± 0.2 ^a^	8.8 ± 0.2 ^a^	1.5 ± 0.2 ^a^	2.7 ± 0.1 ^a^	2.1 ± 0.1 ^a^

The physical parameters 1—length, 2—width, 3—base height, 4—total side height, and 5—total height in the middle, given in cm, were determined according to Figure 1. For each type of sample, in each column, different lowercase letters represent significantly different values (*p* < 0.05).

**Table 5:** Mean values of lightness (L*), red–green (a*), yellow–blue (b*), and total colour difference (ΔE) on the upper side of muffins baked with different drupelets amounts (0, 60, 90, 120) of fresh, freeze-dried, and convective-dried (40 °C) Versailles raspberry fruits from dormancy state.

	Drupelets	L*	a*	b*	ΔE	Colour ^1^
Fresh	0	66.06 ± 5.79 ^a^	6.07 ± 1.35 ^a^	38.34 ± 5.68 ^a^	-	
	60	52.53 ± 2.74 ^b^	2.35 ± 1.02 ^b^	28.61 ± 4.13 ^b^	17.08	
	90	53.57 ± 6.19 ^b^	2.46 ± 0.61 ^b^	26.81 ± 3.89 ^b^	17.37	
	120	52.46 ± 4.01 ^b^	3.19 ± 1.35 ^b^	26.81 ± 2.94 ^b^	18.06	
Freeze-dried	0	66.06 ± 5.79 ^a^	6.07 ± 1.35 ^a^	38.34 ± 5.68 ^a^	-	
	60	68.02 ± 2.44 ^a^	6.06 ± 1.48 ^a^	38.43 ± 5.17 ^a^	0.09	
	90	68.31 ± 1.99 ^a^	4.64 ± 1.54 ^a^	39.62 ± 3.13 ^a^	2.96	
	120	69.24 ± 2.40 ^a^	5.43 ± 1.35 ^a^	41.93 ± 3.07 ^a^	4.84	
Dried at 40 °C	0	66.03 ± 5.79 ^a^	6.22 ± 1.22 ^a^	27.20 ± 5.68 ^a^	-	
	60	52.59 ± 5.51 ^a^	4.27 ± 0.68 ^b^	31.59 ± 2.64 ^a^	14.69	
	90	55.98 ± 6.25 ^a^	4.78 ± 0.89 ^b^	25.03 ± 4.20 ^a^	10.38	
	120	46.18 ± 5.25 ^a^	4.39 ± 1.07 ^b^	35.02 ± 2.85 ^a^	24.01	

^1^ Muffin colour using an online correspondence CIELab parameters program using RGB (red, green, and blue) values [33]. For each type of sample, in each column, different lowercase letters represent significantly different values (*p* < 0.05).

**Table 6:** Mean values of lightness (L*), red–green (a*), yellow–blue (b*), and total colour difference (ΔE) of upper and lower sides of muffins baked with sodium bicarbonate and yeast raising agents with 120 fresh drupelets of Versailles variety from dormancy state.

Raising	Drupelets	L*	a*	b*	ΔE	Colour ^1^
**Upper side**						
Bicarbonate	0	67.01 ± 4.84 ^a,A^	6.63 ± 0.70 ^a,A^	37.11 ± 2.49 ^a,A^	-	
	120	55.98 ± 2.49 ^b,B^	4.78 ± 0.96 ^b,A^	35.03 ± 2.67 ^a,A^	11.37	
Yeast	0	68.56 ± 4.23 ^a,A^	7.92 ± 0.33 ^a,A^	36.70 ± 1.95 ^a,A^	-	
	120	63.91 ± 4.82 ^a,A^	6.32 ± 1.64 ^a,A^	36.66 ± 2.18 ^a,A^	4.91	
**Lower side**						
Bicarbonate	0	46.17 ± 3.22 ^a,A^	15.94 ± 2.04 ^a,A^	36.36 ± 1.94 ^a,A^	-	
	120	38.38 ± 2.04 ^b,B^	14.58 ± 2.38 ^a,A^	26.32 ± 1.95 ^b,B^	10.95	
Yeast	0	47.77 ± 3.40 ^a,A^	18.40 ± 1.10 ^a,A^	37.45 ± 2.20 ^a,A^	-	
	120	48.79 ± 3.34 ^a,A^	17.26 ± 1.17 ^a,A^	36.32 ± 1.95 ^a,A^	2.61	

^1^ Muffin colour using an online correspondence CIELab parameters program using RGB (red, green, and blue) values [33]. In each column, for each type of raising agent, different lowercase letters represent significantly different values (*p* < 0.05), while the uppercase letters represent significantly different values (*p* < 0.05) among the muffins, independently of the raising agent.

## Data Availability

Data are contained within the article and Supplementary Materials.

## Supplementary Materials

The following supporting information can be downloaded at: https://www.mdpi.com/article/10.3390/foods12244443/s1, Figure S1: Scheme of the muffin making procedure, Figure S2: Example of the chromatogram obtained for fresh Pacific Deluxe variety from dormancy state: Cy-3-s: Cyanidin-3-O-sophoroside, Cy-3-gr: Cyanidin-3-O-glucosyl rutinoside, Cy 3-(6″-dg): Cyanidin 3 (6′-dioxalyl-glucoside), Cy 3-(6″-s-gl): Cyanidin-3-(6′-succinyl-glucoside), Figure S3: Images of the muffins using 60, 90, and 120 drupelets of fresh, freeze-dried, and convective-dried raspberry fruits at 40 °C, Figure S4: a) Images of the muffins prepared with different raising agents and with 120 raspberry drupelets: muffins made with sodium bicarbonate (left) and muffins made with yeast (right) and b) muffin pH and correspondence with the raising agent used, Table S1: Tentatively identified phenolic compounds, at 520 nm, in fresh, freeze-dried, and convective-dried (30 °C and 40 °C) raspberry fruits of Pacific Deluxe and Versailles varieties from dormancy state, Table S2: Ingredients and respective quantity used in muffin formulations, Table S3: Mean values of lightness (*L**), red–green (*a**), yellow–blue (*b**), and total colour difference (ΔE) in the lower side of muffins baked with different drupelets amounts (0, 60, 90, 120) of fresh, freeze-dried, and convective-dried (40 °C) raspberry fruits from Versailles variety from dormancy state, Table S4: Physical parameters of muffins baked with sodium bicarbonate and yeast raising agents with 120 fresh drupelets from Versailles variety from dormancy state.
