# Supplementary material for: Quality Characteristics of Raspberry Fruits from Dormancy Plants and Their Feasibility as Food Ingredients
Source: Foods. 2023 Dec 11;12(24):4443. doi: 10.3390/foods12244443 (PMC10742829; doi:10.3390/foods12244443)
Supplement: Supplementary file 1 [file foods-12-04443-s001.zip › foods-2760677-supplementary.pdf]

Quality characteristics of raspberry fruits from dormancy plants and their feasibility as food ingredients

Sílvia Petronilho*, Manuel A. Coimbra, and Cláudia P. Passos*

* Correspondence: silviapetronilho@ua.pt (S.P.) and cpassos@ua.pt (C.P.P.)

Supplementary material

Figure captions:
Figure S1. Scheme of the muffin' making procedure.
Figure S2. Example of the chromatogram obtained for fresh raspberry fruits of Pacific Deluxe variety from dormancy state: Cy-3-s: Cyanidin-3-*O*-sophoroside, Cy-3-gr: Cyanidin-3-*O*-glucosyl rutinoside, Cy 3-(6"-dg): Cyanidin-3-(6'-dioxalyl-glucoside), Cy 3-(6"-s-gl): Cyanidin-3-(6'-succinyl-glucoside).
Figure S3. Images of the muffins using 60, 90, and 120 drupelets of fresh, freeze-dried, and convective-dried raspberry fruits at 40 °C.
Figure S4. a) Images of the muffins prepared with different raising agents and with 120 raspberry drupelets: muffins made with sodium bicarbonate (left) and muffins made with yeast (right) and b) muffins pH and its correspondence with the raising agent used.

Table captions:
Table S1. Tentatively identified phenolic compounds, at 520 nm, in fresh, freeze-dried, and convective-dried (30 °C and 40°C) raspberry fruits of Pacific Deluxe and Versailles varieties from dormancy state.
Table S2. Ingredients and respective quantity used in muffins' formulations.
Table S3. Mean values of lightness (L^*), red-green (a^*), yellow-blue (b^*), and total colour difference (ΔE) in the lower side of muffins baked with different drupelets amounts (0, 60, 90, 120) of fresh, freeze-dried, and convective dried (40 °C) raspberry fruits from Versailles variety from dormancy state.
Table S4. Physical parameters of muffins baked with sodium bicarbonate and yeast raising agents with 120 fresh drupelets from Versailles variety from dormancy state.

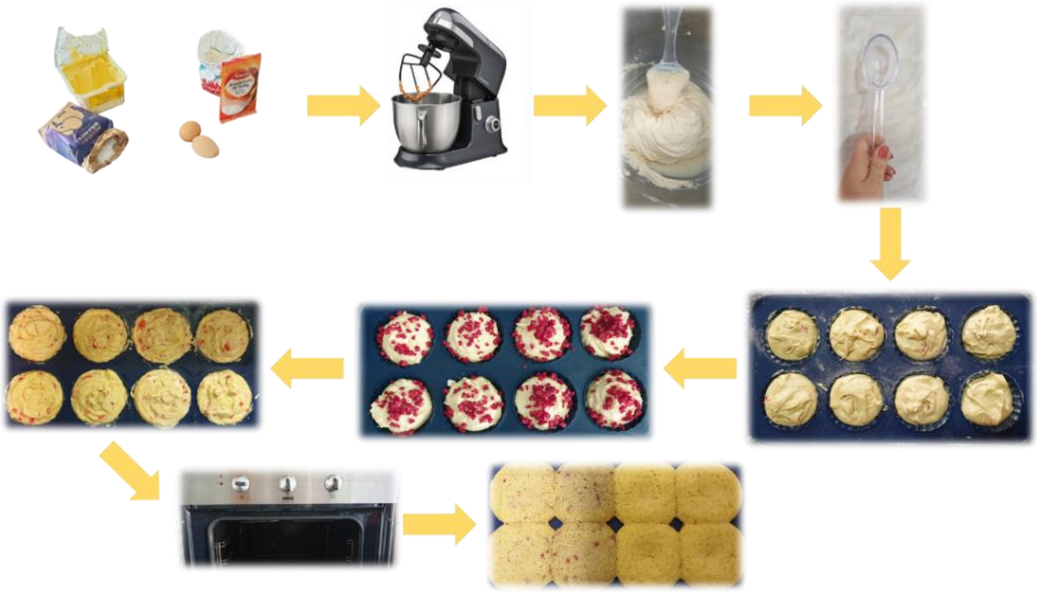

Figure S1. Scheme of the muffin' making procedure.

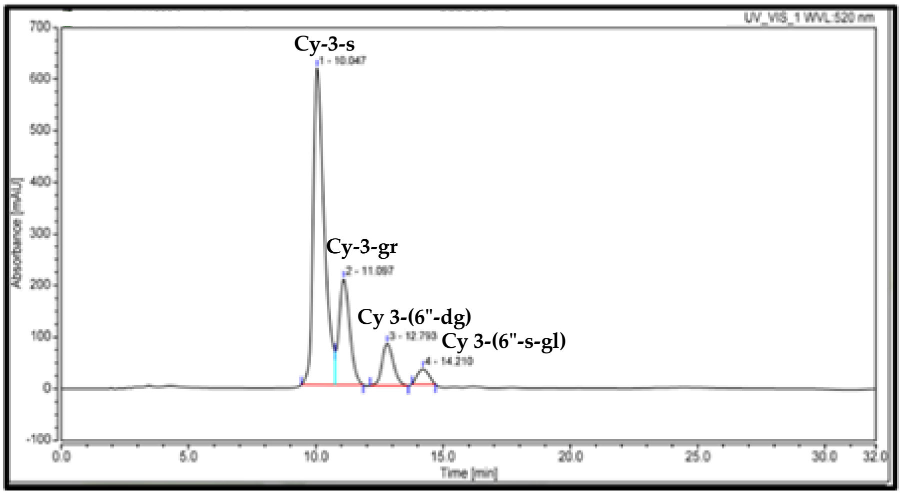

Figure S2. Example of the chromatogram obtained for fresh raspberry fruits of Pacific Deluxe variety from dormancy state: Cy-3-s: Cyanidin-3-*O*-sophoroside, Cy-3-gr: Cyanidin-3-*O*-glucosyl rutinoside, Cy 3-(6"-dg): Cyanidin-3-(6'-dioxalyl-glucoside), Cy 3-(6"-s-gl): Cyanidin-3-(6'-succinyl-glucoside).

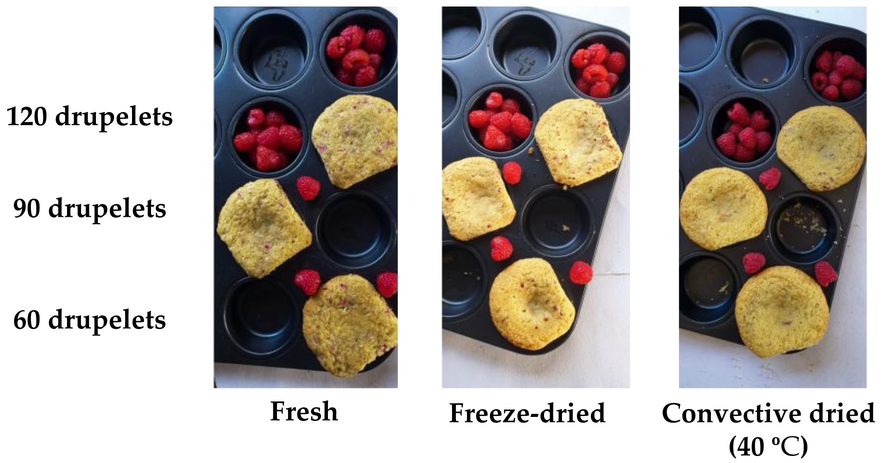

Figure S3. Images of the muffins using 60, 90, and 120 drupelets of fresh, freeze-dried, and convective-dried raspberry fruits at 40 ºC.

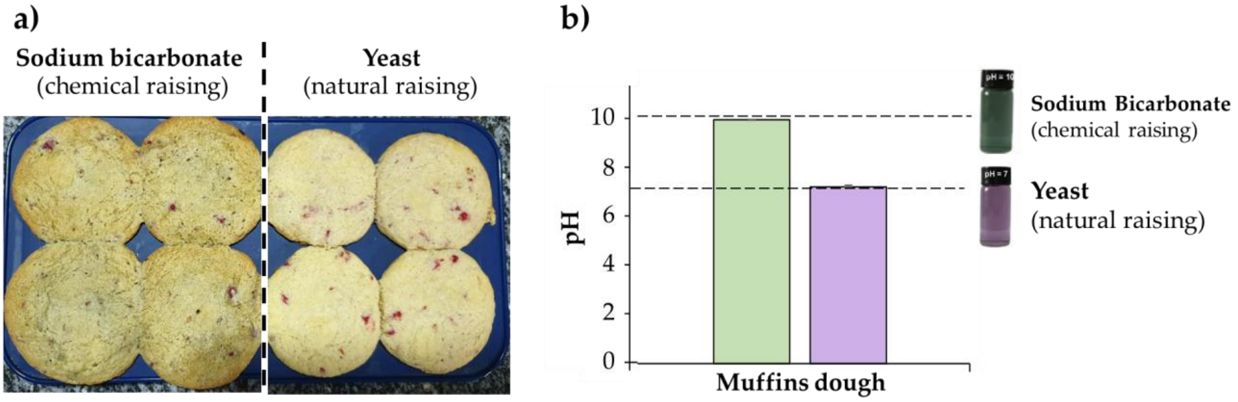

Figure S4. a) Images of the muffins prepared with different raising agents and with 120 raspberry drupelets: muffins made with sodium bicarbonate (left) and muffins made with yeast (right) and b) muffins pH and its correspondence with the raising agent used.

Table S1. Tentatively identified phenolic compounds, at 520 nm, in fresh, freeze-dried, and convective dried (30 °C and 40°C) raspberry fruits of Pacific Deluxe and Versailles varieties from dormancy state.

RT (min)	Compound	MS (*m/z*)	MS^2 (*m/z*)
10.047	Cyanidin-3-*O*-sophoroside	609.38	285.07
11.097	Cyanidin-3-*O*-glucosyl rutinoside	755.42	285.10
12.793	Cyanidin-3-(6'-dioxalyl-glucoside)	593.38	285.16
14.210	Cyanidin-3-(6'-succinyl-glucoside)	771.40	285.10

RT: retention time in minutes; MS: mass spectrum.

Table S2. Ingredients and respective quantity used in muffins' formulations.

Ingredients	Mass (g)
Wheat flour	360
Sugar	270
Butter without salt	200
Mixed egg (egg number)	2
Sodium bicarbonate/Yeast[1]	9
Raspberry (drupelets number)	0, 60, 90, 120

[1]Replacement of the sodium bicarbonate with the same amount of yeast.

Table S3. Mean values of lightness (*L**), red-green (*a**), yellow-blue (*b**), and total colour difference (ΔE) in the lower side of muffins baked with different drupelets amounts (0, 60, 90, 120) of fresh, freeze-dried, and convective dried (40 °C) raspberry fruits from Versailles variety from dormancy state.

	Drupelets	*L**	*a**	*b**	ΔE	Colour[1]
**Muffins downside**						
Fresh	0	47.34 ± 1.97^{a}	14.74 ± 0.76^{a}	33.78 ± 1.34^{a}	-	
	60	49.23 ± 3.53^{a}	13.12 ± 1.97^{a}	34.10 ± 3.37^{a}	2.50	
	90	42.43 ± 2.13^{a}	13.97 ± 1.61^{a}	28.32 ± 6.44^{a}	7.38	
	120	41.37 ± 4.28^{a}	12.43 ± 1.20^{a}	28.12 ± 6.81^{a}	8.54	
Freeze-dried	0	47.34 ± 1.97^{a}	14.74 ± 0.76^{a}	33.78 ± 1.34^{a}	-	
	60	45.16 ± 2.65^{a}	15.35 ± 0.76^{a}	31.72 ± 2.72^{a}	3.06	
	90	45.07 ± 2.84^{a}	15.48 ± 0.54^{a}	31.65 ± 2.41^{a}	3.20	
	120	45.25 ± 4.42^{a}	14.61 ± 0.77^{a}	31.10 ± 3.06^{a}	3.41	
Dried at 40 °C	0	39.48 ± 1.97^{a}	16.40 ± 0.76^{a}	34.40 ± 1.34^{a}	-	
	60	45.45 ± 5.45^{a}	13.67 ± 1.67^{a}	32.38 ± 2.80^{a}	6.87	
	90	45.97 ± 5.97^{a}	12.35 ± 1.35^{a}	32.05 ± 1.73^{a}	8.00	
	120	46.69 ± 6.69^{a}	13.20 ± 1.20^{a}	32.16 ± 2.58^{a}	8.20	

[1] Fruits colour using an online correspondence CIELab parameters program using RGB (red, green, and blue) values (https://www.e-paint.co.uk/convert-lab.asp). ΔE is the colour difference found in the formulation prepared with different amounts of drupelets compared to the corresponding control (without drupelets). For type of sample, in each column, different lower-case letters represent significantly different values ($p<0.05$).

Table S4. Physical parameters of muffins baked with sodium bicarbonate and yeast raising agents with 120 fresh drupelets from Versailles variety from dormancy state.

			Parameters (cm)				
**Raising agent**	**Drupelets**	**Weight (g)**	**1**	**2**	**3**	**4**	**5**
Bicarbonate	0	$38.3 \pm 3.1^{a,A}$	$7.7 \pm 0.3^{a,A}$	$8.6 \pm 0.2^{a,A}$	$1.5 \pm 0.0^{a,A}$	$2.8 \pm 0.3^{a,A}$	$2.0 \pm 0.1^{a,A}$
	120	$40.3 \pm 2.6^{a,A}$	$7.4 \pm 0.3^{a,A}$	$8.2 \pm 0.2^{a,A}$	$1.5 \pm 0.1^{a,A}$	$2.7 \pm 0.3^{a,A}$	$2.2 \pm 0.2^{a,A}$
Yeast	0	$45.0 \pm 2.0^{a,B}$	$7.2 \pm 0.2^{a,A}$	$8.1 \pm 0.4^{a,A}$	$1.5 \pm 0.1^{a,A}$	$2.4 \pm 0.2^{a,B}$	$2.2 \pm 0.3^{a,A}$
	120	$44.0 \pm 1.0^{a,B}$	$7.4 \pm 0.6^{a,A}$	$7.7 \pm 0.3^{a,B}$	$1.5 \pm 0.1^{a,A}$	$2.3 \pm 0.2^{a,B}$	$2.2 \pm 0.2^{a,A}$

The physical parameters 1 - length, 2 - width, 3 – base height, 4 - total side height, and 5 - total height by the middle, given in cm, were determined according to Figure 1. For each type of raising agent, in each column, different lowercase letters represent significantly different values ($p<0.05$), while the uppercase letters represent significantly different values ($p<0.05$) among the muffins, independently of the raising agent.
